# Enhanced peer-review for optimising publication of biomedical papers submitted from low- and middle-income countries: feasibility study for a randomised controlled trial

**DOI:** 10.1192/bjo.2018.89

**Published:** 2019-02-04

**Authors:** Alexandra Pitman, Raphael Underwood, Adam Hamilton, Peter Tyrer, Min Yang

**Affiliations:** Associate Professor, UCL Division of Psychiatry; and Honorary Consultant Psychiatrist, Camden and Islington NHS Foundation Trust, St Pancras Hospital, UK; Trainee Clinical Psychologist, Institute of Psychiatry, Psychology and Neuroscience, King's College London, UK; Editorial Assistant, British Journal of Psychiatry, Royal College of Psychiatrists; and Freelance Editor, UK; Emeritus Professor of Community Psychiatry, Centre for Psychiatry, Imperial College London, Claybrook Centre, Charing Cross Hospital, UK; Professor of Medical Statistics, Department of Epidemiology and Health Statistics, West China School of Public Health, China; and Visiting Adjunct Professor, Swinburne University of Technology, Australia

**Keywords:** Low and middle income countries, randomized controlled trial, feasibility study, peer review, capacity building

## Abstract

**Background:**

Biomedical research from low- and middle-income countries (LMICs) is poorly represented in Western European and North American psychiatric journals.

**Aims:**

To test the feasibility of trialling a capacity-building intervention to improve LMIC papers' representation in biomedical journals.

**Method:**

We designed an enhanced peer-review intervention delivered to LMIC corresponding/first authors of papers rejected by the *British Journal of Psychiatry*. We conducted a feasibility study, inviting consenting authors to be randomised to intervention versus none, measuring recruitment and retention rates, outcome completion and author/reviewer-rated acceptability.

**Results:**

Of the 26/121 consenting to participate, 12 were randomised to the intervention and 14 to the control arms. Outcome completion was 100% but qualitative feedback from authors/reviewers was mixed, with attrition from 5/12 (42%) of intervention reviewers.

**Conclusions:**

Low interest among eligible authors and variable participation of expert reviewers suggested low feasibility of a full trial and a need for intervention redesign.

**Declaration of interest:**

A.P., P.T. and M.Y. are *British Journal of Psychiatry* editorial board members. During this study P.T. was *British Journal of Psychiatry* Editor, A.P. was a trainee editor and A.H. was an editorial assistant.

## Background

The striking underrepresentation of published research from low- and middle-income countries (LMICs) in Western European and North American psychiatric journals has been attributed to low submission rates in the context of the inadequate resourcing of psychiatric research.^1^ Beyond language bias in systematic reviews,^2^ it is suggested that the low frequency of research articles relevant to health problems in resource-poor countries^3^ represents the unconscious bias of journal editors and peer reviewers.^4^

This is thought to be linked to editors' and peer reviewers' views regarding the relevance of research from LMICs, as well as perceptions of the quality of submitted papers, both in methodology and written English. If such unconscious biases do exist this has serious implications for the implementation of evidence-based technical medical interventions in LMIC settings^5^ and has been described as a form of editorial racism.^6^ This term describes any system that systematically discriminates against others on the grounds of race or creed, with or without intention or knowledge, resulting in different outcomes for people from different racial groups.^6^

## Capacity-building

Factors relevant to global inequities in publication of biomedical science include underresourced research infrastructures in LMICs that limit opportunities for supervision and training and hamper the design and conduct of primary research and literature reviews. Pressure to publish in high-impact journals may also necessitate writing in a second language.^7^ Investment in LMIC research infrastructures is a long-term project, but shorter-term solutions have been suggested in the form of capacity-building. These include providing free access to biomedical literature,^8^ active collaborations between resource-rich universities and researchers from high-income countries and journal editors reviewing their editorial practice. Editors can take positive action by diversifying their editorial boards and peer reviewers, providing a more balanced representation of international research, and taking a more collaborative approach over editing methodologically sound papers. Authors whose papers are rejected without review lose the educational opportunity afforded by comments from a thoughtful peer reviewer, with most such rejection letters providing little constructive feedback. Provision of detailed feedback on why a manuscript was rejected, together with suggestions for improvements, provides a means of capacity-building and minimising acceptance bias.

Our objective was to test the feasibility of a randomised controlled trial (Capacity Enhancement in Psychiatric Publications Randomised Intervention Trial, CEPPRIT) to assess the impact of an enhanced peer-review process for rejected papers submitted from resource-limited countries in optimising their chances of publication within an indexed journal. We designed an intervention delivered by experienced peer reviewers, operationalised using the existing editorial system of papers submitted to the *British Journal of Psychiatry*, a psychiatric journal published monthly by the Royal College of Psychiatrists. We developed a trial protocol and report the findings of our feasibility study here; conducted to assess rates of recruitment and retention, and on which to base assumptions about effect sizes for a potential full trial.

## Method

### Study participants

Our inclusion criteria for participation in the feasibility study were: any paper submitted to the *British Journal of Psychiatry* by a first or corresponding author from a LMIC over a 12-month period from 1 November 2011 and rejected without review. We defined LMICs using 2012 World Bank criteria, which classified low-income countries as those including India, Nigeria and China and middle-income countries as those including Brazil, Romania, Bulgaria, Lebanon and Mexico.^9^ On the basis of past submissions, we estimated that 220 papers per year would fulfil these criteria. Submissions that fulfilled inclusion criteria for participation in the trial during the recruitment period were identified by the journal's editorial assistant (A.H.). The corresponding/first author for each eligible paper was sent an email signed by the journal's Editor (P.T.) explaining that the paper had been rejected without review, but inviting them to take part in a randomised trial of enhanced peer review.

### Intervention

Our intervention was allocation to a capacity-building expert reviewer, who had been given the remit of improving the standard of the paper to a level suitable for publication in a biomedical journal. Given the potentially intensive nature of the enhanced peer-review process we wished to select those reviewers most likely to commit to detailed and timely responses. Expert reviewers were recruited in 2011 from the previous year's 128 top-ranking peer reviewers for the *British Journal of Psychiatry,* with reviewers rated annually for volume and speed of reviews. A total of 46 reviewers agreed to participate (36%), on the understanding of being allocated up to two manuscripts each during this study.

### Study procedures

The email inviting eligible authors to take part in the trial explained that authors randomised to the intervention arm would be offered ‘detailed and constructive feedback to improve their papers after they have been rejected for publication’. It was clarified that at any point in this process authors in the intervention arm could resubmit their paper to any journal, and although this could include the *British Journal of Psychiatry*, there was no guarantee of acceptance. In the case of a resubmission, our study protocol was that the paper would be allocated to a different handling editor. The email explained that those allocated to the control group would receive no further contact, with the expectation that they would submit their paper elsewhere.

Papers for which the author consented to participate in the trial were randomised from a remote location by our trial statistician to either the intervention or control (no intervention) arms on a 1:1 ratio using block randomization with randomly varied blocks of four, six and eight.

Each paper allocated to the intervention arm was assigned consecutively to an expert reviewer. Authors randomised to the intervention received an email informing them that the reviewer they had been allocated would email them up to six times over a maximum period of 6 months, providing comments that ‘may involve suggestions about: a possible journal to send the paper to, rewriting slightly or extensively, doing further analyses or expanding the work a little, or possibly abandoning the project in favour of a somewhat different one’.

Intervention arm reviewers were provided with the name of their allocated author and instructed to, thorough peer review, help the author improve their manuscript and suggest which journals might be better suited to their study. They were asked to format their input such that their initial email provided detailed peer-review comments on the submitted manuscript, with subsequent emails responding to queries or revisions.

### Outcomes

Our measures of feasibility were recruitment rates (response to sampling invitation) and retention rates (as reported by reviewers). We measured acceptability by contacting allocated reviewers to explore their experiences of contact with authors, and to measure their authors' retention in the study.

We followed up participating authors to explore their experiences of participating. We also contacted non-responders to ask them their reasons for not responding, explaining ‘We are particularly concerned that the invitation might have been perceived as patronising or unnecessary as you were already experienced in getting papers published, but it would be helpful to know the reasons, whatever they are’.

As this was a feasibility study, we did not conduct a sample size calculation, but collected data on prospective outcomes for a full trial. We measured, at 3 years after initial submission: publication in an indexed journal; publication in any (indexed/unindexed) journal (binary outcomes) and impact factor (continuous outcome, for published papers). We were unable to estimate an effect size for group difference on these measures because of the lack of previous studies. However, one goal of this study was to provide parameters on which to base assumptions about effect sizes for a potential full trial.

Outcomes were collected by searching PubMed, Google Scholar and Scopus by author and approximations of the submission title, to determine whether it had been published within 3 years of submission to the *British Journal of Psychiatry*, whether the journal was indexed and the impact factor. Given the large number of non-responders to the initial invitation to participate, a *post hoc* decision was made to add these as a second control group, measuring publication outcomes in a similar way. Conducting such a search was felt to be ethical given that publication was a matter of public record and that no identifying details would be given in this paper. We therefore collected outcomes for the 92 non-responders too.

### Ethics

We did not seek ethical approval for this feasibility study because the initiative was developed as a potential editorial service improvement for submitting authors and the readership. The protocol reflected a variation of the existing submission process for papers submitted to the *British Journal of Psychiatry*, which was considered and approved by the Royal College of Psychiatrists Publication Management Board in May 2011.

### Statistical analysis

We used χ^2^ tests to compare groups on the proportion of submissions that had been published in an indexed journal within 3 years of submission. We used logistic regression to test for an association between the intervention and whether a submission was published in an indexed journal within 3 years of submission, and whether published in any journal, adjusting for country of origin of corresponding/first author. We used linear regression to test for an association between the intervention and mean impact factor in the subset of those submissions that were published in a journal with an impact factor.

Our comparison of outcomes was between the intervention and control arms. To increase statistical power, we also performed a *post hoc* comparison adding non-responders to controls, such that our comparison was intervention versus controls plus non-responders. This also helped determine whether adding those with a lead time advantage (resubmitting to another journal immediately rather than waiting for reviewer's comments) affected outcomes. We analysed data on an intention-to-intervene basis.

## Results

### Response

**Fig. 1:**
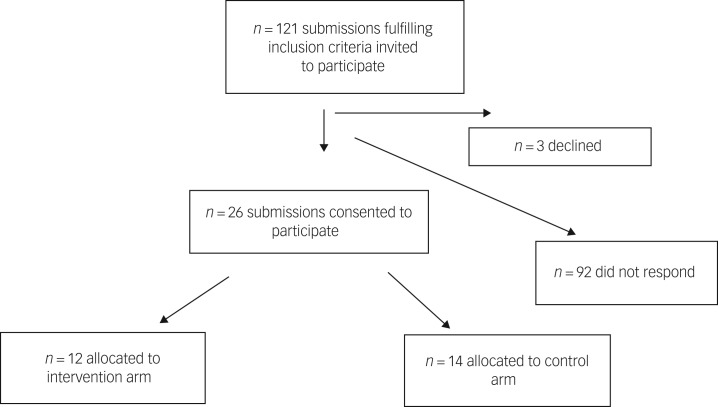
Participant flow.

The recruitment period was extended beyond 12 months to 22 months, because of the lower number of eligible papers than anticipated and the number of non-responders. Between 8 November 2011 and 9 September 2013 a total of 121 papers fulfilling inclusion criteria were identified, and each of the 121 corresponding authors was invited to participate in the trial (Fig. 1). We stopped at 22 months for reasons of resources, falling short of the target 220 eligible papers anticipated.

Of the 121 invited, 3 corresponding/first authors declined to take part in the trial and 92 did not respond to the invitation, representing a response rate of 24% (29/121) and a consent rate of 22% (26/121). The 26 consented submissions were allocated to either the intervention (*n* = 12) or control arms (*n* = 14). Twelve reviewers were allocated 1:1 to the 12 submissions in the intervention arm.

### Intervention delivery

Follow-up contact with these reviewers revealed that 5/12 (42%) had never contacted their allocated author, despite repeated reminders from editorial staff. These five submissions were analysed on an intention-to-intervene basis. Of the remaining seven allocations, two reviewers received no response after contacting their allocated author. Five reviewers maintained correspondence with their allocated authors, and two received a revised manuscript following their peer-reviewer comments. Feedback from reviewers on their experiences of the interactive peer-review process was mixed (see Appendix).

Three participating authors responded to follow-up inviting comments on their experience of participating. One (control) could not remember having been randomised, and the other two provided comments revealing mixed experiences (Appendix). Of 92 non-responders, 10 responded to our follow-up inviting comments on reasons for not participating. Their reasons for not taking part were coded using a basic classification as: preferring a fast-track assessment (*n* = 1); perceiving the email as patronising (*n* = 1); lack of perceived utility (*n* = 1); being too busy to take part because of exams/moving institution (*n* = 2); being on extended leave (*n* = 1) and not having received the initial email (*n* = 4).

### Sample characteristics

Among those consenting to take part in the study (*n* = 26) the country of origin represented most frequently was India (*n* = 8) followed by China (*n* = 5). For non-responders the highest proportion was China (*n* = 32) followed by India (*n* = 25). Of those refusing participation (*n* = 3), two papers were submitted from India and one from China. Of those included in the analysis (non-responders and consenters), China represented the highest proportion of submissions (*n* = 40), followed by India (*n* = 33). Based on frequencies we derived a six-category variable to denote country of origin: India, China, Nigeria, other Indian subcontinent, Middle East, and other (Table 1).

**Table 1:** Publication outcomes for the three arms of the feasibility study 3 years after submission

Allocation	Country of origin *n*			Published in an indexed journal within 3 years of submission *n* (%)			Published in any journal within 3 years of submission, *n* (%)			Impact score of published papers	
	India	China	Other^a^	Yes	No	χ^2^ (*P*)	Yes	No	χ^2^ (*P*)	Mean (s.d.)	*F* (*P*)
Intervention (*n* = 12)	2	4	6	6 (50)	6 (50)		8 (67)	4 (33)		1.63 (1.01)	
Control (*n* = 14)	6	1	7	7 (50)	7 (50)		9 (64)	5 (36)		1.39 (1.35)	
Non-responders (*n* = 92)	25	35	32	53 (58)	39 (42)		57 (62)	35 (38)		2.07 (2.05)	
Total (*n* = 118)	33	40	45	66 (56)	52 (44)		74 (63)	44 (37)		1.98 (1.88)	
Test statistics						0.476 (0.788)			0.064 (0.969)		0.660 (0.520)

a. Category 3: Nigeria (*n* = 8); Category 4: Pakistan (*n* = 6), Bangladesh (*n* = 1); Category 5: Iran (*n* = 12), Turkey (*n* = 2), Iraq (*n* = 5), Egypt (*n* = 2), Category 6: Brazil (*n* = 4), Columbia (*n* = 1), Mexico (*n* = 1), Romania (*n* = 1), Cuba (*n* = 1), Jordan (*n* = 1).

### Publication outcome measurement

We measured outcomes for submissions in all three arms. Our sieve of three different search engines, allowing for variations in title wording, appeared to be a robust method of identifying publication in an indexed journal. Overall for the three arms, 56% of submissions had been published in an indexed journal within 3 years of submission, and 63% had been published in any indexed journal (Table 1). Non-responders had the highest proportion of journals published in an indexed journal (58%), followed by those in the intervention arm (50%) and the control arm (50%). For articles published in any journal, the intervention arm had the highest proportion (67%), followed by the control group (64%) and non-responders (62%). The results of χ^2^-testing showed no significant group differences in proportions on either outcome.

When we tested for an association between publication outcome and country of origin, we found no significant differences in relation to publication in an indexed journal, or in any journal. However, papers published by Chinese authors had a significantly higher mean impact factor (supplementary Table 1 available at https://doi.org/10.1192/bjo.2018.89).

Our multivariable logistic and linear regression analyses found no evidence to support an effect of the intervention on any of the three outcomes after adjusting for country of origin. This was the same whether comparing the intervention with controls (Table 2), or to all those not receiving the intervention (non-responders and controls, Table 3), suggesting that adding those with a lead time advantage in resubmitting to another journal had no effect on outcomes.

**Table 2:** Results of multivariable models testing for the effect of the intervention on outcomes (main analysis: intervention versus control)

Comparison group	Primary outcome: paper published in indexed journal, AOR^a^ (95% CI)	Secondary outcome: paper published in any journal, AOR^a^ (95% CI)	Secondary outcome: impact score of published paper, estimated mean difference^a^ (s.e.) *P*
Control (*n* = 14)	1	1	0.594 (0.526)
Intervention (*n* = 12)	0.730 (0.111–4.808)	0.763 (0.121–4.831)	−0.660 (0.483) 0.172

AOR, adjusted odds ratio; s.e., standard error.

a. Adjusted for country of origin.

**Table 3:** Results of multivariable models testing for the effect of the intervention on outcomes (intervention versus control + non-responders)

Comparison group	Primary outcome: paper published in indexed journal, AOR^a^(95% CI)	Secondary outcome: paper published in any journal, AOR^a^ (95% CI)	Secondary outcome: Impact score of published paper, estimated mean difference^a^ (s.e.) *P*
Control + non-responders (*n* = 106)	1	1	0.761 (0.386)
Intervention (*n* = 12)	0.778 (0.226–2.680)	1.071 (0.274–4.184)	−0.432 (0.600) 0.471

AOR, adjusted odds ratio; s.e., standard error.

a. Adjusted for country of origin.

## Discussion

### Main findings

Our feasibility study identified problems with the acceptability of this enhanced peer-review approach. Our response (24%) and consent rates (22%) were low, suggesting that a full trial would have similar problems with recruitment. We also identified problems with implementation fidelity, such that only 7 of 12 peer reviewers had contacted their allocated author. While only three of those authors had sent back a revised manuscript, this did not preclude other authors having submitted their revised manuscript elsewhere. These findings of low levels of interest among authors, together with the mixed experience of reviewers, suggested low feasibility of the intervention. Our comparison of publication outcomes (albeit underpowered) also provided no evidence to support the effectiveness of enhanced peer review as a means of improving the chances of publication. We did note that among published papers, those by Chinese authors had the highest mean impact factor. This may be because of financial publication incentives for Chinese researchers,^10^ as well as its comparative economic growth.^11^

Our qualitative feedback from non-responders suggests that our low response and consent rates may have been because some of the invitations had been missed (perhaps in junk mail), some authors had felt it more efficient to submit directly to another journal and that the invitation had been experienced as patronising. The latter is most likely to have been the case for authors from rapidly advancing economies such as Brazil, China and India. Development indicators are dynamic and since this study commenced, the 2014 United Nations Human Development Report classified China as a high-income country.^11^

Regardless of country of origin, there exists a general problem that any suggestion of affirmative action in editorial decisions could be seen as both patronising and discriminatory.^1^ This serves to reinforce inequalities and is counterproductive. Even those who did not perceive the invitation as patronising may have declined it under pressure to publish quickly, submitting to other journals with a greater likelihood of providing peer reviewers' comments and of acceptance. By its nature the intervention involved a longer and more detailed feedback process, and authors may not have perceived this time cost to be worth the anticipated benefits, particularly with no guarantee of paper acceptance.

The largest group of non-responders in our study were from China, a country where authors are under great pressure to publish papers in indexed journals to gain career promotion or monetary awards at work. This would have dissuaded involvement in a trial on the basis of these time costs. Communication with journal editors is not commonplace in Chinese biomedical culture, and some authors may have perceived negatively the prospect of repeated communication with reviewers during the trial. Pressure to publish quickly may also have placed these authors at risk of predatory publishers, which disproportionately trap LMIC authors.^12^

Almost 50% of the intervention arm did not receive the intervention, reflecting low engagement of even the most active peer reviewers, the majority of whom were editorial board members. It is possible that this reflected competing time pressures to submit grant applications and research papers, in the context of teaching and clinical responsibilities and a high frequency of requests for peer review. The burden on peer reviewers may not have been realistic, but our approach to choosing peer reviewers may also not have been optimal. Predictors of quality of peer review in general medical journals include younger age of reviewer, training in epidemiology or statistics and longer time taken to review (up to 3 h), with reviews by editorial board members conversely rated as poor quality.^13^ It is possible that recruitment of younger reviewers might have improved the quality of the intervention, albeit perhaps less acceptable to authors invited to participate. Anonymised peer review with correspondence channelled through editorial assistants would overcome this issue, but at the expense of increased pressure on journal staff.

This final issue lies at the crux of how best to deliver capacity-building interventions to improve the international dissemination of research from LMICs. Many biomedical journals are edited by academics offering their time *pro bono*, with paid staff also under pressure to achieve targets. The time and resources required for enhanced peer review are vulnerable to cuts and competing priorities, unless specifically funded by bodies interested in providing such a service. It might be argued that instead of funding such short-term solutions, funds would be better invested upstream and over the longer term, for example in training early career researcher in LMICs in methodology and report-writing.

### Results in the context of other studies

We are not aware of other trials of any similar intervention. Other approaches to building capacity in higher education and research in LMICs include investing in training in preparation of research proposals and publications, but this has not been formally evaluated. In some countries, university research funding formulae are based on numbers of publications, with the concern that this drives higher volumes of lower-quality papers. Where the impact of such formulae has been evaluated in high-income settings, this has not been found to affect quality of research output negatively.^14^ However, this is likely to be because of separate pressure from grant-awarding bodies to publish in higher-impact international peer-reviewed journals.

### Strengths and limitations

This was a pragmatic trial that recruited papers routinely submitted to, and rejected from, a high-impact psychiatric journal, thus reflecting current practice in editorial decision-making. The trial responded to the need for capacity-building in research from LMICs and placed minimal resource burden on participating authors, apart from requiring them to wait up to 6 months while corresponding with reviewers. It did, however, place a burden on peer reviewers, as reflected in the high proportion of those who did not implement the intervention. Recruiting early-career researchers as peer reviewers could improve the intervention while providing a valuable training opportunity. As the invitation to participate may have been perceived as patronising to authors, a redesign of this wording should also be considered.

Our use of randomisation was intended to solve baseline differences but given low numbers in a small feasibility study we acknowledge the possibility of imbalance. Although we adjusted for country of origin, no adjustment was made for other baseline characteristics (such as gender of first/corresponding author; whether affiliated to a university; whether the submitting author had a Masters/PhD level qualification; whether a high-income country author was involved), as we assumed that randomisation would solve baseline differences. Our study was not designed to investigate whether or not institutional racism operates in journal editors' decision-making.^4^ Inherent to such an investigation would be an assessment of paper quality, which was beyond the remit of this study.

### Policy implications

Our results do not support the use of enhanced peer review in this form for submissions from LMICs as a means of optimising their chances of publication, whether in an indexed journal or not. However, a redesign of the intervention could be considered, following consultation with LMIC authors, and addressing issues such as the wording of the invitation to participate, the recruitment of reviewers most likely to deliver enhanced peer review, and the realistic administrative time resources of journal staff. Uptake might also be improved by sending the invitation soon after the rejection email, rather than as part of it, and including some preliminary verdict on how the paper could be improved, to provide something immediately and tangibly positive in the invitation.

Other means should also be sought for achieving the aim of improving the representation of international research published in psychiatric journals. Journal editors should consider how they can change practice to achieve their goals of global legitimacy^4^ and widening their readers' perspectives on health.^5^ One option is setting quotas for research articles relevant to resource-poor countries.^3^ Publishers should consider redistributive solutions to the prohibitive costs of publishing in Open Access journals for institutions unsupported by research funding councils. These include Oxford University Press's Developing Countries Initiative^15^ to set reduced or free publication costs, presumably subsidised by high-income country submissions.

Upstream investment in research infrastructure is an avenue recommended by the World Health Organization, and global charitable foundations such as the Wellcome Trust may have a role in driving this. Examples include delivery of training in research methodology and scientific writing, mentoring opportunities, research collaborations and improved access to mental health research publications.^16^ A partnership system between research departments in high-income countries and LMICs would provide opportunities for researchers to receive reciprocal feedback on the design of research protocols and on the style of draft manuscripts, reinforced by video-conferencing. Such interventions should follow good practice on high-income country–LMIC collaboration, to avoid parasitism and encourage mutually productive partnerships.^17^ Capacity-building workshops are also an opportunity for journal editors to meet with researchers to provide constructive feedback on common reasons for rejection. Where previously used within Europe our experience is that these have been well-received and have improved the quality of submissions.

Global foundations also have a role in improving the dissemination of research from LMICs, using digital technology to penetrate new readerships with the aim of improving mental health provision in all countries of the world. A combination of efforts at all these levels has the potential to achieve the World Psychiatric Association's goal of improving mental health provision in all countries regardless of local research resources, as well as improving LMIC research representation in biomedical journals.

## Supplementary material

For supplementary material accompanying this paper visit http://dx.doi.org/10.1192/bjo.2018.89. click here to view supplementary material

## References

[1] Patel V, Sumathipala A. International representation in psychiatric literature: survey of six leading journals. Br J Psych 2001; **178**: 406–9. 10.1192/bjp.178.5.406 11331553

[2] Delgado-Rodriguez M, Llorca J. Bias. J Epidemiol Community Health 2004; 58: 635. 15252064 10.1136/jech.2003.008466 PMC1732856

[3] Obuaya C. Reporting of research and health issues relevant to resource poor countries in high-impact medical journals. Eur Sci Editing 2002; 28: 72–7.

[4] Horton R. Medical journals: evidence of bias against the diseases of poverty. Lancet 2003; 361: 712–3. 12620731 10.1016/S0140-6736(03)12665-7

[5] Barr D, Fenton L, Edwards D. Politics and health. QJM 2004; 97: 61–2. 14747619 10.1093/qjmed/hch015

[6] Tyrer P. Combating editorial racism in psychiatric publications. Br J Psych 2005; 186: 1–3. 10.1192/bjp.186.1.1 15630115

[7] Jiang X, Borg E, Borg M. Challenges and coping strategies for international publication: perceptions of young scholars in China. Stud High Educ 2015; 42: 428–44.

[8] Ajuwon GA, Olorunsaye JO. Knowledge, access and usage pattern of HINARI by researchers and clinicians in tertiary health institutions in south-west Nigeria. Afr J Med Med Sci 2013; 42: 97–106. 23909100

[9] World Bank. World Bank Data and Statistics. World Bank, 2012.

[10] Jiang X, Borg E, Borg M. Challenges and coping strategies for international publication: perceptions of young scholars in China. Stud High Educ 2017; 42: 428–44.

[11] Fantom NJ, Serajuddin U. The World Bank's Classification of Countries by Income (English). *Policy Research working paper; no. WPS 7528* World Bank Group, 2016.

[12] Clark J. Letter to the Editor - predatory journals: bad for all but especially authors from low and middle income countries. Acta Méd Port 2018; 31: 184–5. 29790475 10.20344/amp.10489

[13] Black N, van RS, Godlee F, Smith R, Evans S. What makes a good reviewer and a good review for a general medical journal? JAMA 1998; 280: 231–33. 9676665 10.1001/jama.280.3.231

[14] van den Besselaar P, Heyman U, Sandstrom U. Perverse effects of output-based research funding? Butler's Australian case revisited. J Informetrics 2017; 11: 905–18.

[15] Oxford University Press. Developing Countries Initiative. Oxford Univeristy Press, 2018 (https://academic.oup.com/journals/pages/librarians/developing_countries).

[16] World Health Organization. Galvanising Mental Health Research in Low- and Middle-Income Countries: Role of Scientific Journals - a Joint Statement issued by Editors of Scientific Journals Publishing Mental Health Research and Department of Mental Health and Substance Abuse. WHO, 2004.

[17] Hedt-Gauthier B, Airhihenbuwa CO, Bawah AA, Burke KS, Cherian T, Connelly MT, Academic promotion policies and equity in global health collaborations. Lancet 2018; 392: 1607–9. 30496066 10.1016/S0140-6736(18)32345-6

