# Enhanced peer-review for optimising publication of biomedical papers submitted from low- and middle-income countries: feasibility study for a randomised controlled trial – CORRIGENDUM

**DOI:** 10.1192/bjo.2019.17

**Published:** 2019-03-08

**Authors:** Alexandra Pitman, Raphael Underwood, Adam Hamilton, Peter Tyrer, Min Yang

**Keywords:** Low and middle income countries, randomized controlled trial, feasibility study, peer review, capacity building, erratum

In the original publication of this article the article title and number were incorrectly stated.

The correct citation details are as follows:

Pitman A, Underwood R, Hamilton A, Tyrer P, Yang M. Enhanced peer-review for optimising publication of biomedical papers submitted from low- and middle-income countries: feasibility study for a randomised controlled trial. *BJPsych Open* 2019; 5(2): e20. https://doi.org/10.1192/bjo.2018.89.

This has now been updated in the original article online.

The publisher and author sincerely apologise for these errors.
