# Supplementary material for: Enhanced peer-review for optimising publication of biomedical papers submitted from low- and middle-income countries: feasibility study for a randomised controlled trial
Source: BJPsych Open. 2019 Feb 4;5(2):e20. doi: 10.1192/bjo.2018.89 (PMC6401541; doi:10.1192/bjo.2018.89)
Supplement: Supplementary file 1 [file S2056472418000893sup001.docx]

**Appendix – Supplementary Table 1 – Outcomes of publication by country of origin**

| Country code* | N | Paper published in indexed journal  % | Paper published in any journal  % | Impact factor of published papers  Mean ± SD |
| --- | --- | --- | --- | --- |
| 1 | 33 | 48.5 | 57.6 | 0.73±1.28 |
| 2 | 40 | 67.5 | 67.5 | 3.22±2.04 |
| 3 | 8 | 62.5 | 75.0 | 1.22±1.22 |
| 4 | 7 | 42.5 | 57.1 | 1.82±1.28 |
| 5 | 21 | 47.6 | 66.7 | 1.21±1.05 |
| 6 | 9 | 55.6 | 55.6 | 1.82±1.75 |
| Sig (P value) |  | χ2= 4.129  P=0.531 | χ2= 1.691  P=0.890 | F = 5.815  P=0.000 |

* Category 1=India, Category 2 = China, Category 3: Nigeria (n=8); Category 4: Pakistan (n=6), Bangladesh (n=1); Category 4: Iran (n=12), Turkey (n=2), Iraq (n=5), Egypt (n=2), Category 6: Other - Brazil (n=4), Columbia (n=1), Mexico (n=1), Romania (n=1), Cuba (n=1), Jordan (n=1).
